# Gene Duplication and the Genome Distribution of Sex-Biased Genes

**DOI:** 10.4061/2011/989438

**Published:** 2011-09-05

**Authors:** Miguel Gallach, Susana Domingues, Esther Betrán

**Affiliations:** Department of Biology, University of Texas at Arlington, P.O. Box 19498, Arlington, TX 76019, USA

## Abstract

In species that have two sexes, a single genome encodes two morphs, as each sex can be thought of as a distinct morph. This means that the same set of genes are differentially expressed in the different sexes. Many questions emanate from this statement. What proportion of genes contributes to sexual dimorphism? How do they contribute to sexual dimorphism? How is sex-biased expression achieved? Which sex and what tissues contribute the most to sex-biased expression? Do sex-biased genes have the same evolutionary patterns as nonbiased genes? We review the current data on sex-biased expression in species with heteromorphic sex chromosomes and comment on the most important hypotheses suggested to explain the origin, evolution, and distribution patterns of sex-biased genes. In this perspective we emphasize how gene duplication serves as an important molecular mechanism to resolve genomic clashes and genetic conflicts by generating sex-biased genes, often sex-specific genes, and contributes greatly to the underlying genetic basis of sexual dimorphism.

## 1. Introduction

Sexual dimorphism occurs in species that produce differentiated sexes, most commonly, males and females. This implies that a single genome carries the information to generate two well-differentiated organisms characterized by sex-specific tissues, behaviors, and physiologies. As a consequence, the genome experiences different selective pressures when carried by males or females, and, therefore, for a single species, two optimal genomes will be selected [1]. This situation generates a conflict between sexes of the same species, known as sexual antagonism (SA), because the optimal genome for one of the sexes (i.e., the genome that confers maximum fitness to one of the sexes) is detrimental in the other sex, and *vice versa*. Understanding how sexual dimorphism occurs, what are the evolutionary forces driving sexual antagonism, and what mechanism can finally or temporarily resolve the conflict are important biological questions. 

Currently, the existence of high-throughput technologies permits the study of gene expression on a genomic scale and the identification of genes that are differentially expressed between the sexes of a given species (i.e., sex-biased or sex-specific genes). These studies are helping evolutionary biologists understand how sexual dimorphism is attained. One of the most interesting results that came out of these studies has been the discovery that sex-biased genes are not randomly distributed in the genome of organisms with heteromorphic sex chromosomes (called XY or ZW chromosomes when males or females are the heterogametic sex, resp.; [2–6]). Heteromorphic chromosomes [7–10] are an exciting subject of study due to their unique biological features, such as hemizygosis in the sex carrying the heteromorphic sex chromosomes, dosage compensation (DC) and meiotic sex chromosome inactivation (MSCI). These biological features have been postulated to play a central role in the distribution of sex-biased genes as well as in sexual conflict. 

The advent of the first genome sequences also revealed that gene duplication has played an important role in the distribution of sex-biased genes. Betrán et al. [11] analyzed the whole gene set of *D. melanogaster* and showed that retrogenes (i.e., genes originating from the retrotranscription of a parental mRNA molecule) are not randomly distributed throughout the genome. Instead, the authors found that there is a significant excess of retrogenes located on the autosomes that originated from X-linked parental copies (i.e., an excess of X to autosome duplication pattern), with any other pattern being significantly underrepresented. Importantly, an additional feature of these retrogenes is that their expression tends to be male-specific while the respective parental genes have wide expression patterns [11]. Subsequent analyses have confirmed these patterns not only in other species [12–15], but also for DNA-mediated duplicates [14, 15], suggesting that the same evolutionary forces are probably favoring the relocation and sex-biased expression of duplicated genes. After these evidences, new empirical and theoretical studies are attempting to integrate gene duplication in the sexual conflict resolution. 

In this perspective, we: (1) introduce methods for detecting sex-biased gene expression on a genomic scale and discuss the degree to which sexes and tissues contribute to gene expression differentiation, (2) describe analyses that have revealed the nonrandom distribution of sex-biased genes in distantly related species and discuss how much of these patterns can be explained by DC, MSCI and SA hypotheses, and (3) highlight the importance of gene duplication as a driver of the genome distribution of sex-biased genes and outline the recently proposed role of gene duplication as a means to resolve genomic clashes (e.g., escape from MSCI or DC; [12–16]), adaptive conflict, and intralocus sexually antagonistic conflict [17]. 

To favor a more friendly reading, we include two boxes with aside information that complement the text. In Box 1, we review the biological features of heteromorphic sex chromosomes, including hemizygosity of the X and Z chromosomes, DC, MSCI, and location of SA variation. In Box 2, we highlight examples of the multiple ways in which sex-biased genes can originate through gene duplication.

**Box 1. The Biology of the Sex Chromosomes**

**Figure 1:**
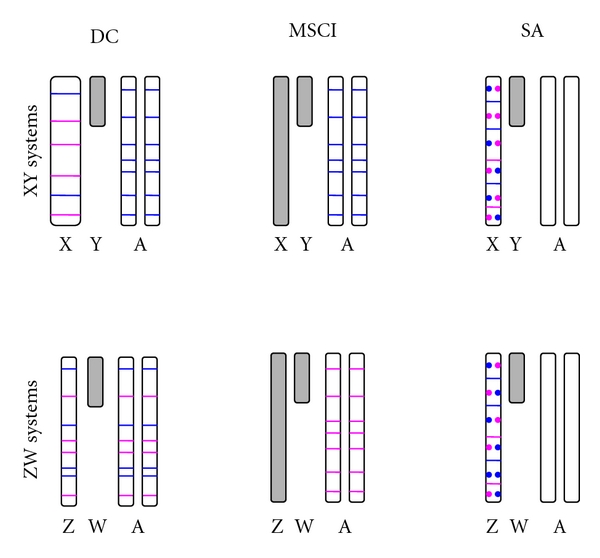
Predictions of the standard DC (Model II and Model V in Table 1), MSCI (Model I in Table 1) and SA (Model VII in Table 1) models on the location of sex-biased genes. In XY systems with DC, the X chromosome is hypertranscribed to equal the level of expression of both autosomal and X chromosome genes in females (not shown). Note that the X chromosome is wider (i.e., hypertranscribed) than the autosomes. Under this hypothesis, there will be physical constraints evolution of male-biased expression for highly expressed genes located on the X chromosome on the (blue lines), while this constraint does not exist for genes evolving female-biased expression (pink lines) or even for male-biased genes expressed at low levels. Consequently, most male-biased genes will be located on the autosomes. Because the ZW system do not have dosage compensation, male-biased genes will be detected on the Z chromosome, but a correction must be made, with the expectation then being that male- and female-biased genes will be equally distributed among the chromosomes. MSCI exists in both XY and ZW systems. Because most sex-biased genes are gonad specific, male- and female-biased genes are predicted to be preferentially located outside of the X and Z chromosomes, respectively; otherwise, they would be inactivated in the gonads. Sexual antagonism predicts that the X and Z chromosomes will carry most of the sexually antagonistic alleles (blue circles for genes beneficial to males and detrimental to females; pink circles for genes beneficial to females and detrimental to males), which has been confirmed in *Drosophila*. Under the classical model [27], resolution of this conflict would be achieved through the evolution of sex-biased and, optimally, sex-specific expression of the antagonistic gene. Therefore, the X and Z chromosomes are predicted to hold the most sex-biased genes. Gray is used to represent heterochromatin or inactivation of the X/Z chromosomes during MSCI.

In many organisms, sex is determined by the presence or absence of a pair of heteromorphic sex chromosomes. This pair of chromosomes can be XY or ZW when males or females are the heterogametic sex, respectively [7–10]. According to the theory of sex chromosome evolution, sex chromosomes were once a pair of equivalent autosomes that evolved into sex chromosomes after one of the homologs acquired a dominant factor for sex determination [18]. Two main processes are believed to drive sex chromosome morphological differentiation, although the speed and extent at which they occur can vary [10, 19]. First, the chromosome bearing the sex-determining gene degenerates as a consequence of the lack of recombination between the homologs [10, 20–23]. Different processes, such as Muller's ratchet, background selection, and selective sweeps, are probably involved in the degeneration of the Y/W chromosome, the importance of which has been discussed in several papers [10, 20–23]. Second, as the Y/W chromosome erodes, strong selective pressures will favor the generation of a compensatory regulatory mechanism of gene expression to balance functional aneuploidy in the heterogametic sex [24–26]. This mechanism is known as dosage compensation (DC; Figure 1), and it is attained in different ways in distant taxa.

For instance, in flies, DC results in an overexpression of the X chromosome in males; in worms, the expression of the two overexpressed X chromosomes in hermaphrodites is reduced by half, and there is overexpression of the single X chromosome of males; finally, female mammals inactivate one of the two X chromosomes and, like males, overexpress their unique active X chromosome (see [24–26, 28, 29]). These DC systems make the male to female and the X to Autosome expression ratios about the same [30, 31].

The independent evolution of DC mechanisms in distantly related XX/XY organisms strongly suggest that the same evolutionary forces might favor the evolution of this compensatory mechanism, not only in XY but also in ZW systems. However, several recent experiments have ruled out the existence of global DC mechanisms acting on the Z chromosomes of birds and Lepidoptera [32–35]. In addition, Prince et al. [36] recently described another interesting case: in the beetle *Tribolium castaneum*, the X chromosome in males is hypertranscribed such that the X to autosomes ratio is equal to 1, as expected when the compensation occurs in the heterogametic sex. However, the two X chromosomes in females are also hypertranscribed, resulting in an X to autosome ratio equal to 1.5 in this sex. The authors argue that these results probably reflect the absence of a general mechanism in females that inhibits or counterbalances the DC system working in males [36], as would be observed in Drosophila, mammals, or nematodes [30]. Altogether, these results indicate that females might be less vulnerable than males to the deleterious effects of the heterogamy [31] and that the concept of dosage compensation as universal necessity on heterogametic sexes must be reevaluated.

In addition to the well-known processes of Y chromosome degeneration and DC, it has been clear for some time that the X chromosome is inactivated in the male germline of eutherian [37–40] and metatherian mammals [41, 42] (i.e., MSCI). The occurrence of X chromosome inactivation in the male germline has also been observed in worm [5, 6, 43]. However, there has been some debate about the existence of MSCI in the Drosophila male germline [44–50]. A recent study of ZW inactivation in birds revealed that MSCI also occurs in heterogametic females [51]. Therefore, and in contrast to DC, MSCI is likely a more general phenomenon of sex chromosomes in the heterogametic sex (Figure 1). Many hypotheses have been proposed to explain the existence of MSCI. Recently, it has been revealed that meiotic silencing of unsynapsed chromatin (MSUC), a mechanism to silence selfish elements and unsynapsed (i.e., unpaired) DNA in order to prevent aberrant chromosome segregation, might be the basis of MSCI [39, 52–54].

An inevitable consequence of the Y/W chromosome degeneration is a state of hemizygosity in the heterogametic sex. This implies that recessive mutations linked to the X/Z will be exposed to selection in XY and ZW individuals. In addition, X/Z chromosomes will spend two thirds of their time in the homogametic sex and only one third in the heterogametic sex. These two circumstances have important consequences from a population and evolutionary point of view, especially for sexually antagonistic genes. In this perspective, we use the term sexually antagonistic gene or simply sexual antagonism (SA), to describe a gene where the two segregating alleles are selected in opposite directions in males and females (i.e., intralocus sexual conflict; [55]), unless otherwise stated. Theory predicts [27] that new SA alleles will increase in frequency and are more likely to remain polymorphic in the population when they are located on the X/Z chromosome than when they are on the autosomes. Hence, a dominant allele linked to the X that is beneficial to females but detrimental to males will spread through the population because the allele will spend two thirds of its time in females and only one third of its time in males. On the contrary, a recessive allele that is beneficial to males but detrimental to females will increase in frequency because it will be always affected by positive selection in hemizygous males but hidden from negative selection in heterozygous females [27]. The inverse rationale can be applied for ZW systems. Under such circumstances, modifiers of the expression have been proposed to evolve in order to reduce (and optimally inhibit) the expression of the SA gene in the harmed sex, resolving the sexual conflict and allowing the fixation of the antagonistic allele ([27]; Model VII in Table 1). From this, it is often assumed that the genomic location of sex-biased genes is a good indicator of the location of past SA genes [56, 57]. We say past because once the gene is sex biased, the antagonism is resolved.

In summary, two important predictions come from this model [27]: (1) because it is easier for SA alleles to increase in frequency when they are located on the X/Z chromosome, these chromosomes will carry most intralocus SA variation and (2) because SA is resolved through the evolution of sex-biased expression of the antagonistic loci, then most sex-biased genes will be also located on the X/Z chromosomes. However, there is now empirical evidence that sex-biased genes are not a good proxy for SA genes (see [89] and discussion in the text).

These biological features of sex chromosomes contribute to explain the location of genes with sex-biased expression and the genetic architecture of sexual dimorphism. However, as we will describe in the following sections, the evolution and distribution of sex-biased genes present several particularities that cannot be satisfactorily explained by any of the aforementioned biological phenomena unless gene duplication is introduced in the models (see several models in Table 1 and details in the text).

## 2. Sex-Biased Gene Expression

The development of high-throughput technologies has allowed the study of sex-biased gene expression on a genomic scale. Thanks to these technologies, we can measure the level of expression of all the genes in each sex and determine which genes are differentially expressed. Analyses of expressed sequence tags (ESTs), serial analysis of gene expression (SAGE), DNA microarrays, and, in recent years, massive parallel sequencing technologies are the methods most often used to quantify gene expression on a genomic scale [90]. Notably, most important advances in understanding genome-wide patterns of expression and their evolution derive from microarray technology. DNA microarrays are platforms containing series of microscopic spots with specific target sequences (i.e., oligonucleotides or cDNAs). Under stringent conditions, the probes (e.g., cDNAs or genomic DNA) are labeled with a fluorophore and will hybridize specifically with the target sequences such that the intensity of fluorescence emitted from a spot is proportional to the amount of the hybridizing probe [91]. Through this procedure, one can compare the relative expression of thousands of genes between tissues, individuals or strains (e.g., brain versus testis; larvae versus adults; males versus females; population A versus population B) in a single set of experiments. Although microarray technology has important limitations, many of which will fade away with the application of massive parallel sequencing technologies [90, 92], microarrays still have sufficient sensitivity as to detect small differences among samples [93].

Microarray analyses have been successfully employed in many different studies [93], but only a handful of these studies have focused on detecting sex-biased expression, mostly in Drosophila [94]. A gene is considered to have sex-biased expression when its level of expression is significantly different between sexes. Obviously, the significance will depend on the experimental design as well as on the statistical test and the arbitrary cutoff applied. This is an important problem, as can be observed from the heterogeneous results obtained from different studies (Table 2), that reveals the necessity to standardize procedures in order to facilitate comparisons among studies [95]. As a general convention, a gene or transcript is considered sex biased when the normalized log signal quotient for the sexes differs by at least twofold [94]. This commonly accepted arbitrary cutoff [96] can be justified when the measurements for a significant proportion of genes are within twofold of the measurements from control hybridizations [2]. 

Most studies suggest that about half of Drosophila genes are sex biased, with most expression differences between sexes occurring in the gonads ([2, 61, 95–98]; see also Table 2). In addition, males often contribute more to the number of sex-biased genes and almost exclusively to the number of sex-specific genes [99, 100] as females express highly in ovaries many of the genes used for basic cellular functions. Recently, Prince et al. [36] analyzed more than 98% of the predicted coding transcripts in *T. castaneum* and found that 20% of them had differential expression between sexes, with 58% being female biased. However, at a 2-fold cutoff 75% of the sex-biased genes were male biased. On the other hand, Reinke et al. [5] found that about 29% of the elements tested in the nematode *Caenorhabditis elegans* (representing the 94% of the nematode's gene set) showed differentiated expression at twofold cutoff and that most of the expression divergence was generated by tissues in the gonads. However, in the case of *C. elegans*, hermaphrodites make a larger contribution to sex-biased genes than males. In the mosquito *Anopheles gambiae*, females also contribute more than males to the number of sex-biased genes [101]. It is likely that the differences in the number and types of sex-biased genes between mosquitoes and Drosophila reflect the important differences in behavioral and immune traits between the sexes in mosquitoes [101].

Sex-biased expression has been also studied in a few vertebrates, arriving at similar conclusions to those described for Drosophila. Microarray experiments in chicken and mice showed that in these organisms most of the sex-biased expression occurs in the gonads and that the contribution of males to the number of genes that show expression divergence between sexes is higher than that of females [102, 103]. Even in organisms without heteromorphic sex chromosomes or without sex chromosomes (i.e., Xenopus and Zebrafish), this holds true [104, 105].

In summary, males contribute more than females to the expression divergence between sexes in most analyzed species. In addition, further analyses have proven that male-biased expression contributes highly to interspecific gene expression divergence, indicating that male-biased genes also make a significant contribution to divergence among species in flies [3, 95, 97, 106] and mammals [107, 108]. Hence, the ratio of interspecific divergence in gene expression to intraspecific variation is significantly higher for male-biased genes than for female-biased and unbiased genes [106]. This result is consistent with positive selection operating over expression divergence in male-biased genes, while purifying or neutral selection would be operating on female-biased and unbiased genes. In agreement with this result, Conallon and Knowles [71] showed that the evolution of male-biased expression is mostly an active change; that is, male-biased expression is achieved by increasing the expression in males relative to females (for additional comments see [72, 109]).

The differences in the evolutionary patterns of male-biased compared to female-biased and unbiased genes are also observed at the protein sequence level. Male-biased genes have higher evolutionary rates (i.e., higher Ka/Ks ratios) than female-biased genes and unbiased genes that have been interpreted as adaptive evolution [84, 107, 110]. In addition, sex-biased expression and Ka/Ks ratios correlate with expression divergence in Drosophila [97, 111–114] and mammalian species [115], suggesting that a common selective force underlies all these processes [97]. This fast evolution at the sequence and expression level and the fact that most male-biased genes are testis-specific [2, 90, 95, 97, 98, 116] suggest that once expression testis-specific expression is achieved, genes may be released from pleiotropic constraints and, consequently, be able to evolve more quickly responding to the specific selective pressures of the particular tissue at the expression and sequence level than more widely expressed genes [116]. In agreement with this idea, a recent study revealed that the sex-specific expression in reproductive organs correlates with a high rate of gene evolution, not explained by a narrow pattern of expression [117].

**Figure 2:**
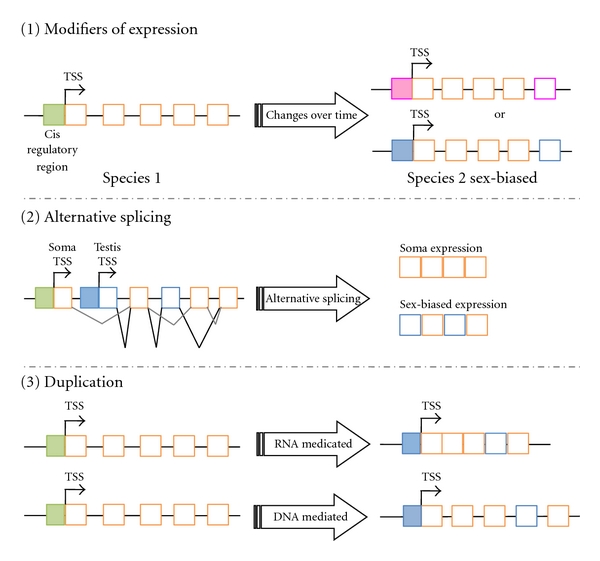
Sex-biased expression can be acquired in three different ways. (1) Changes in the cis-regulatory regions or in *trans*-acting factors can lead to a gene that is more highly expressed in females (pink) than in males or more highly expressed in males (blue) than in females. (2) A gene can have alternative transcripts through alternative splicing. One of those transcripts might be sex-biased in expression. This sex-biased expression can often, though not always, be detected by standard methods (e.g., microarray analyses) if it leads to higher expression in one sex, but it may require direct analysis of alternative transcripts. (3) Gene duplication can lead to the creation of sex-biased genes (e.g., a male-biased gene) through RNA- or DNA-mediated gene duplication. Gene duplication might create one or two sex-biased genes. For details of the proposed models, see Table 1. In the three instances, the exons might specialize. Gene expression patterns are shown with filled boxes and exons with open boxes. Green and orange refer to expression in both sexes. Pink refers to female-specific expression or specialization of an exon. Blue refers to male-specific expression or specialization of an exon.

Given that the origin of most gene expression divergence among species involves changes in male-biased gene expression and that this divergence might be conceivably driven by positive selection, it is crucial to understand the underlying evolutionary processes and forces driving these patterns. There are three different mechanisms that can lead to the evolution of sex-biased or sex-specific gene or isoform expression (Figure 2): (1) evolution of modifiers of the expression (i.e., *cis*- or *trans*-regulatory changes [118]), (2) generation of sex-specific transcriptional variants (with or without coding-exon duplication [76, 77]), and (3) generation of sex-specific genes by gene duplication [11–13, 15, 58, 64].

The evolution of modifier(s) of gene expression may turn a widely expressed gene into a sex-biased gene. The relative contribution of *cis-* and *trans*-regulatory changes to the evolution of expression divergence is a topic under study [119]. Analysis of allele expression in the F1 individuals resulting from crossing different lines of Drosophila allowed the estimation of the relative contribution of *cis*- and *trans*-regulatory changes to the expression divergence in this genus. Under this framework, many studies have shown that *cis*-effects explain more intraspecific [120, 121] and interspecific changes [122–124] than *trans*-effects do (but see also [125]). However, while in a few cases *cis*-effects are clearly implicated in the regulation and expression divergence between sexes [118], its implication on a genomic scale is not known. *Cis-* and *trans*-regulatory changes towards sex-biased expression might be unlikely in single copy housekeeping genes, as these changes might be detrimental in depriving one of the sexes of a basic molecular function [17, 64].

The generation of sex-specific transcriptional variants may allow the acquisition of sex-specific protein isoforms (i.e., sex-specific alternatively spliced variants) without modifying the pattern of expression and/or function of the original isoform (i.e., the other alternatively spliced variant). This molecular level of sexual dimorphism has not been widely investigated. Research in Drosophila suggests that between 22% and 32% of the transcriptional variants are sex specific [126, 127]. A fraction of these variants are known to contribute to sex determination, but others are active only at the very end of the regulatory cascade, leading to a sexually dimorphic phenotype [126, 127]. Sex-specific splice variants are most abundant in the testis, they are also common in the head [127] and likely have important functions because they are highly conserved in Drosophila [127]. Sex-specific transcriptional variants have also been observed in mammals and often in the testis [77, 128].

Finally, gene duplication can also generate sex-specific genes. This is another way of generating sex-biased genes without modifying the expression pattern of the original (parental) gene [17, 64]. While particular examples of sex-specific duplicate genes have been accumulating for a long time (e.g., [58, 129]), including genes duplicated onto the Y chromosome [130], interest in the mechanisms behind this has increased since the first studies showing the nonrandom distribution of retrogenes and their tendency to be expressed specifically in males [11, 13]. Similar to the tissue-specific expression, gene duplication could also lead to the release from the pleiotropic constraints on the original function of the parental gene. Hence, gene duplication would allow for the evolution of new functions in one of the paralogs, while the other copy keeps its broad expression and original functions [17, 131]. In the following sections, we review the main studies in the last few years on the genomic location of sex-biased genes and the role of gene duplication in the evolution and genomic distribution of these genes.

## 3. Genomic Location of the Sex-Biased Genes

Many studies in XY systems have shown that X chromosomes in Drosophila [2–4], and nematodes [5, 6] have a significant underrepresentation of male-biased genes. In mammals, the distribution is highly dependent on cell type. For instance, there is an excess of male-biased premeiotic and postmeiotic genes on the X [62, 132] but the contrary is true for genes expressed during meiosis [63]. In addition, some X-linked genes seem to be overexpressed in brain in males and females [26]. In the particular case of Drosophila, chromosomal rearrangements have been critical in demonstrating that the compositional nature of the X chromosome is not just an historical peculiarity of this chromosome. Instead, evolutionary forces must be operating on the sex chromosomes such that newly formed X chromosomes develop the same composition as the original X chromosome to which it was fused [4, 133]. For instance, in the *Drosophila pseudoobscura *lineage, ancestral Muller elements A (the current XL arm, homologous to the X chromosome in *D. melanogaster*) and D (the current XR arm, homologous to the autosomal arm 3L in *D. melanogaster*) were fused, generating a new metacentric X chromosome about 10–18 million years ago [134, 135]. Interestingly, this originally autosomal arm underwent demasculinization during its evolution to a neo-X chromosome. There is currently an underrepresentation of male-biased genes on that chromosomal arm, something not observed in species where this chromosome is and autosome [4]. These observations strongly suggest that evolutionary forces might be favoring demasculinization of X chromosomes [2, 4]. It seems, however, that this trend might be only a feature of old male-biased genes since a trend for young male-biased genes to originate through gene duplication or “de novo” on the X chromosome has been observed [136–138]. These new male-biased genes are either translocated, lost or may lose the sex-biased expression as the trend reverses for older male-biased genes. For older male-biased genes, an excess is observed in the autosomes consistent with an overall excess of male-biased genes on autosomes in some species (i.e., Drosophila). 

The patterns observed for the Z chromosomes, however, are not conclusive or consistent across species. This lack of consistency might reflect that the studies are often not done on whole organism but on particular tissues. For instance, analyses in chicken first suggested that male-specific genes expressed in brain are significantly overrepresented on the Z chromosome [139, 140]. In contrast, male-specific genes expressed in the testis appeared to be randomly distributed and female-specific genes expressed in brain and ovary were observed to be significantly underrepresented on the Z chromosome [139, 140]. Subsequent publications have contradicted these findings. More recently, Mořkovýský et al. [141] published another analysis showing that oocyte-genes are overrepresented on the Z chromosome. In addition, other studies suggest that testis-specific genes are overrepresented on the silkworm's Z chromosome, another organism with a ZW system [142]. Recently, Mank et al. analyzed the expression patterns genome-wide during chicken development and concluded that contrary to the prediction of current models [27], most sex-biased genes are located on autosomes [74]. 

Regardless of the difficult interpretation of the results obtained in organisms with ZW systems, disentangling the causes that shape the distribution of sex-biased genes in genomes containing heteromorphic sex chromosomes is one of the most intensely active fields of research related to this topic. The actions of DC, MSCI, and SA on sex chromosomes have been suggested as a way to explain the nonrandom distribution of sex-biased genes. 

DC systems hypertranscribe the X-linked genes in somatic male cells to raise the expression level to that of the autosomes and the two X chromosomes in females (reviewed in [30]). Because male-biased expression evolves in Drosophila by increasing the level of expression in males relative to females [71], evolving male-biased expression (i.e., higher expression in males) might be more difficult for highly expressed genes when located on the already hypertranscribed X chromosome than it would be for autosomal genes. 

Vicoso and Charlesworth [72] tested this hypothesis by comparing the genomic locations of male-biased genes with different levels of expression and confirmed that highly expressed genes located on the X are less likely male biased than lowly expressed genes. Moreover, in agreement with this prediction, X-linked genes that are not dosage compensated (i.e., not bound by the dosage compensation complex) have a higher probability of showing male-biased expression than dosage compensated genes [73]. Therefore, under this hypothesis, the demasculinization of the X chromosome in Drosophila would be a consequence of a physical limitation on the ability of dosage compensated genes to increase their level of expression ([72]; Model II in Table 1). It has also been suggested that this effect is attributable to DC interfering with the evolution of male-biased expression (i.e., further upregulation of DC genes in males [73]; Model IV.A in Table 1). The most obvious limitations of the first model is that it only explains the X chromosome deficit for highly expressed male-biased genes, and a limitation of both is that most male-biased genes are testis-specific [2, 61], a tissue where DC is reportedly absent or at least not mediated by the dosage compensation complex [29, 45, 143]. Additionally, these models cannot explain the defeminization (i.e., the significant underrepresentation of female-biased genes) of the chicken Z chromosome, because, as we explained above, this organism has not evolved any dosage compensation mechanism.

An additional model (Model IV.B in Table 1) has just recently been proposed [50]. The authors suggest that the hyperacetylated state of the X chromosome in males due to DC reduces the capability of other regulatory mechanisms to repress tissue-specific X-linked genes in other tissues. According to this hypothesis, highly tissue-biased (testis-specific, as well as any other tissue-specific) genes will be selected to be located out of the X chromosome to assure its silenced state in other tissues (ovary or somatic tissues). This would explain the paucity of male-biased genes on the X as many of them are testis-specific genes. Interestingly, they also found that ovary is the only tissue where DC is not interfering with the evolution of tissue-specific expression. Further analyses are needed to understand the different effect of DC in male- and female-biased expression.

As described in Box 1, MSCI is likely a general property of heterogametic sexes. In nematodes [144], flies [47, 49], but see [50], mammals [37], and chicken [51], an early X or Z chromosome inactivation has been observed early in meiosis. During this stage of the spermatogenic/oogenic process, X/Z-linked genes are repressed, whereas autosomal genes are actively transcribed. The MSCI model postulates that it would be beneficial to duplicate an X/Z-linked gene to an autosome if it was required during MSCI. Because most sex-biased genes are gonad specific, MSCI could explain the demasculinization or defeminization of the X or Z chromosomes, respectively (Model I in Table 1). However, although this hypothesis could potentially explain a large fraction of the sex-biased genes, as most sex-biased genes are testis-biased [61], an important observation threatens this hypothesis. The rationale proposed by Parisi et al. [2] was that if MSCI were the sole cause of the X chromosome demasculinization, then we would expect a random distribution of somatic male-biased genes. Contrary to this expectation, demasculinization of the X chromosome is also observed in gonadectomized flies [2].

Finally, it has been both predicted [27] and observed [89, 145] that the X and Z carry the most intralocus SA polymorphisms. The resolution of this conflict following the classic model could explain the distribution of sex-biased genes [27], because the resolution is predicted to occur through the evolution of sex-biased expression, as introduced above. In particular, when the effects of a new antagonistic allele are mostly dominant or partially dominant, then female-beneficial (male-deleterious) alleles will increase in frequency on the X chromosome and these genes will evolve into female-biased genes. Similarly, male-biased genes will only have a good chance to evolve from SA genes in autosomes, explaining the relative demasculinization of the Drosophila X chromosome [2–4]. The inverse rationale can be applied in chicken to explain the defeminization of the Z chromosome ([139, 140] but see [141]). However, this prediction stands on the dominance effects of alleles, because when the antagonistic effects of a new allele are recessive, then male-beneficial (female-deleterious) alleles will increase in frequency on the X chromosome and will evolve into male-biased genes [27].

However, this hypothesis that might apply to some instances, also presents serious difficulties (Model VII in Table 1). First, X chromosomes hold the most intralocus SA variation (at least in Drosophila; [89, 145]), but, contrary to what the classical model predicts, the X and Z do not accumulate most sex-biased genes in Drosophila, nematodes and chicken [2, 4, 5, 56]. Second, contrary to predictions of the SA model, sex-biased expression seems to evolve frequently by increasing rather than decreasing the expression in one of the sexes [71]. Third, intralocus SA does not seem to be resolved but persists instead [78, 89]; probably because modifying the expression of certain genes (such as a single copy housekeeping gene) might not be as easy as proposed by the model when they are also needed to perform basic, not sexually differentiated, cellular tasks [17]. In those instances, the transient resolution of SA conflict might occur by gene duplication of the SA allele (see below; Model X), but, given that the broadly expressed gene will still be broadly expressed, SA can reappear (i.e., persist [17]). 

In summary, the general conclusion from analyses performed in nematodes, flies and mammals is that there is a significant paucity of male-biased genes on the X chromosomes in these organisms [2–6, 63]. However, in the case of organisms with heterogametic females, the data are much more limited, and published results have been contradictory. Finally, although several hypotheses have been postulated, none of them can completely explain the distribution of sex-biased genes, and it is not known what amount of bias may be explained by each of the hypotheses. There might not be one model that fits all the data [14] but the role of gene duplication might be more important than previously acknowledged and could have multiple roles outside of that postulated in MSCI hypothesis (e.g., replacing the parental gene). We review the data supporting this in the next section and in Box 2 and remark how gene duplication likely has an unforeseen, multifaceted, and important role in the resolution of the X-related clashes, adaptive conflicts and sexual conflicts generated by the unique biology of this chromosome and sex-related selective pressures.

## 4. The Contribution of Gene Duplication to Sex-Biased Expression: Resolution of Gene Conflicts

Past analyses of retroposed copies of genes have revealed that gene duplicates escape the X chromosome and acquire male functions, often in the male germline [11–13, 16]. This pattern, often called the out-of-the-X pattern, is concomitant with sex chromosome formation in the human lineage [16, 60] and has been observed in other lineages, including mammals and flies, and in the parallel retroduplication of the same duplicated genes in closely related species [12, 16, 146], all of which suggest a shared underlying selective pressure. Unfortunately, analogous studies in chicken have not been fruitful due to the low number of retrogenes in the genome of this species, which is probably caused by the absence of the appropriate type of non-LTR retrotransposons [147].

To discern between mutation and selection as the driving force behind the out-of-the-X pattern observed in retrogenes, Emerson et al. [13] looked at retropseudogenes in the human genome. These authors found that retropseudogenes (i.e., nonfunctional duplicates which are supposed to evolve neutrally, and thus reflect mutational biases) do not show an out-of-the-X pattern, supporting the action of positive selection. However, this procedure cannot be applied in Drosophila, because its genome does not have enough retropseudogenes [12], and therefore, other evidence has to be gathered to uncover any mutational biases in this species. In one analysis, it was found that the out-of-the-X pattern is not explained by the insertional biases recently described for retrotransposable elements [148] that allegedly encode the machinery used for retrotransposition [149]. In *D. melanogaster*, transposable element (TE) insertions, and by extension retrogene insertions are affected by several factors, including recombination, genome compactness, gene expression, and the presence of coexpressed genes clusters [148]. Unlike retrogenes, the number of TE insertions on the X chromosome was higher than expected revealing that the pattern of retrogene distribution cannot be explained by the insertional biases of TEs and suggesting that selection could be a driving force. In another analysis, Vibranovski et al. [15] postulated that if the biased relocation pattern observed for retrogenes was simply a mutational process, we would expect a random distribution for relocated duplicates arising from other mutational mechanisms. Analyses of DNA-mediated duplicates instead showed relocation patterns similar to those observed for retrogenes, that is, an excess of X-to-autosome relocations and male-biased expression of the relocated genes [14, 15]. Studies of patterns of duplications and relocations (duplication with loss of the parental gene) that include not only retrogenes but also DNA-mediated duplications in Drosophila [4, 14, 15] have confirmed that retrogenes are duplicated on autosomes more often than expected. In addition, these studies have also uncovered that in neo-X chromosomes male-biased genes often get relocated to autosomes, mainly through DNA-mediated duplications [14].

In light of these results, it has been suggested that gene duplication is a mechanism that allows X-linked genes to overcome DC or MSCI and achieve male-biased expression (i.e., escape from genomic clashes, MSCI or DC; Table 1). Supporting these ideas, Bachtrog et al. [73] found that the probability that an X-linked gene will generate a relocated copy on an autosome is higher for compensated than noncompensated genes [73]. In addition, autosomal-linked retrogenes originated from X-linked parental genes are more often highly expressed in meiosis, while the respective parental genes seem to be inactivated [16, 49, 73]. Note that these two hypotheses state that escape from the X chromosome is a consequence of DC or MSCI. 

Interestingly, there are some proposed SA models that involve gene duplication. For instance, the SAXI hypothesis (Model VIII in Table 1) suggests an alternative and conceptually different explanation [79]. This hypothesis states that rather than causing relocation of genes from the X chromosome, MSCI is a consequence of the continuous duplication of SA genes from the X to the autosomes. This process would end with the accumulation on the X chromosome of female beneficial antagonistic genes that are detrimental in spermatogenesis. Consequently, MSCI evolved to avoid the expression of these X-linked genes that would be detrimental in male gonads. In other words, SA is the evolutionary force that drives demasculinization of the X chromosome and germline X inactivation. Wu and Xu's paper not only suggest an explanation for the out-of-the-X pattern but also states that gene duplication is a way of resolving sexual antagonism, an idea that was suggested earlier ([80, 81]; Model IX in Table 1). These duplicative models have been recently developed further to try to explain the location of sex-biased genes ([150]; Model IX in Table 1). In these models, the resolution of the intralocus sexual antagonism by gene duplication has been proposed to occur through the creation of a male-specific and a female-specific duplicate gene (Model IX in Table 1). 

All the aforementioned hypotheses, although satisfactory in some instances, cannot cope with the total complexity of this phenomenon (Table 1). For instance, DC cannot explain the complementary expression between parental and testis-specific duplicated genes or the way relocated genes evolve [64–69, 85]; MSCI cannot explain why relocated male-biased genes, which supposedly replace the parental function in the germline, can have a different function, evolve under positive selection or eventually become lost [65–69]; the SAXI hypothesis cannot explain why the export of genes out of the X chromosome is still ongoing and why SA in the X chromosome persists. In addition, recent results present additional challenges to these models [64] by demonstrating that the relocation patterns may vary depending on the type of genes that are being analyzed and that a big fraction of testis-specific genes have been duplicated genes from one autosome to another. Some recent models of the resolution of the intralocus sexual antagonism by gene duplication and the generation of a male-specific and a female-specific gene predict that accumulation of female-biased genes on the X chromosomes and male-biased genes on the autosomes is independent of the dominance effects and can account for the observed autosomal locations of male-biased genes [150]. However, these models do not completely fit the data because most sex-specific genes are male-specific genes derived from broadly expressed genes or sex-specific genes duplicated from other sex-specific genes [4, 11–13, 15, 16, 64, 82–85, 94, 97, 151–153].

Recently, a new duplicative model has been suggested [17, 64]. Gallach et al. [64] studied the duplication patterns of Drosophila mitochondrial genes encoded in the nucleus and found an extreme rate of duplicate relocation for both, RNA- and DNA-mediated duplicates. Interestingly, 83% of the relocated genes evolve at higher rates than their respective parental genes and are expressed only in the testis. Importantly, they also found a significant excess of autosome to autosome relocations [64]. The authors concluded that a particular SA model (Model X in Table 1) was the most satisfactorily explanation for their results and suggested that gene duplication might be an important mechanism for resolving intralocus SA because of the high potential for relocated genes to develop male-specific expression [154–156]. The high potential of relocated genes to develop male-specific expression was proposed to be the result of both insertional biases close to germline genes that facilitate male-specific expression together with selection [64].

Based on these observations, Gallach and Betrán [17] have suggested a new duplicative model for the resolution of intralocus SA (Model X in Table 1). According to this model, relocation and testis-specific expression of the antagonistic allele will result in the parental gene keeping its original function and expression patterns (i.e., wide expression in males and females) with the antagonistic allele fixed in the population as a new gene with testis-specific functions. This model has the power to help interpret multiple observations. It can explain why the X chromosome holds the most sexually antagonistic alleles but is not the place where the conflict is resolved; it can explain why the gene export persists as well as the significant excess of X to autosome duplications; it can also explain why an excess of autosome to autosome duplications is observed for certain sets of genes [64]. In agreement with this hypothesis, there are data that indirectly indicate that male-biased genes originate more often through gene duplication than unbiased or female-biased genes [84, 94, 97, 151]. 

The contribution of gene duplication to sex-biased expression might also come from the duplication of a preexisting sex-biased gene and the driving force could be, in this case, not intralocus sexual conflict but interlocus sexual conflict [55]. It is now clear that a large fraction of sex-biased genes may result from gene duplication of preexisting sex-biased genes [82, 84, 85, 152, 153]. This has occurred multiple times in some gene families (e.g., female reproductive proteases or accessory gland proteins [83–85]). These are sex-specific genes that interact with the other sex and positive and diversifying selection have been proposed to act on those genes as a consequence of strong sexual selection [82, 83]. This process is different from the model above that proposes that the resolution of intralocus sexual antagonism occurs by gene duplication, because the parental genes are already sex specific. Therefore, the model that would fit these data (Model XI in Table 1) is a model that combines positive selection and balancing selection maintaining segregating variation in sex-biased genes with gene duplication; that is, there is balancing selection or adaptive conflict due to diversifying selection and the resolution of this genetic conflict by gene duplication [157]. In the particular cases of the female reproductive proteases and accessory gland proteins, the recurrent action of positive and diversifying selection might lead to a particular dynamic process that involves an increase in gene number (i.e., creation of a gene family) but also eventual pseudogenization of some genes [83–85].

Interestingly, in these last duplicative models (Model X and Model XI in Table 1), there is observed (or assumed) preexisting allelic variation in the parental gene resulting from strong diversifying or sexually antagonistic selection that promotes the gene duplication ([157]; see also model XII in Table 1) and the same selective pressures might persist in the form of positive selection or specialization of the duplicated gene [17]. Finally, the high turnover with respect to gene loss of male-biased genes [84, 97] likely leads to an underestimation of the impact of gene duplication as a means to generate sex-biased genes through these models.

The extent to which each model in Table 1 can explain the genomic location of sex-biased genes is lineage-dependent and some of these models deserve further investigation in many lineages. However, it is clear, as reviewed in this section and highlighted by the examples in Box 2, that gene duplication has an important role in the origin of sex-biased genes. The role of gene duplication could potentially be very large if we consider that the rate of new gene per generation is known to be very high, as revealed by new genomic data [158]. Consistent with this view, recent data from Drosophila suggest that the adult male transcriptome contains more genes of recent origin than the adult female transcriptome indicating that new genes have a role as sex-biased genes in Drosophila [159]. The role of gene duplication in creating sexually dimorphic genes is likely to hold even in organisms without sex chromosomes (e.g., Zebrafish) as the adult transcriptome appears to be young in this species as well [159]. Note that only a small fraction of the models in Table 1 will apply to genomes without sex chromosomes.

**Box 2. Examples of the Generation of Sex-Biased and Sex-Specific Genes through Gene Duplication**

**Figure 3:**
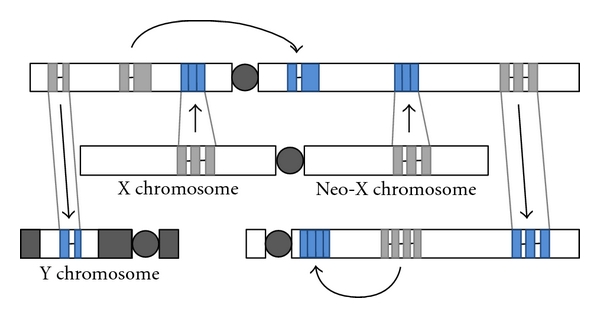
Gene duplication to the Y, out of the X and neo-X chromosomes and between autosomes, often leads (with or without the loss of the parental gene) to sex-biased expression (often male tissue-specific expression; blue). Sex-biased expression (in blue) appears to evolve often as long as the duplication involves relocation. Both RNA-mediated and DNA-mediated gene duplication appear to contribute to these effects.

Gene duplication plays a major role in evolution, because it can increase the dose of a gene, partition the function of a gene or create a new function [160, 161]. Functional data have accumulated revealing that gene duplication patterns contribute greatly to sexual dimorphism in genomes with heteromorphic sex chromosomes, as introduced in the text. In this Box, we highlight some relevant examples of gene duplications, with or without relocation (Figure 3).

**Gene Duplications onto the Y Chromosome**

Duplication of genes to the Y has been observed in mammals, flies, and even in plants [86, 88, 162, 163]. These studies suggest that relocation onto the Y chromosome is one way this chromosome avoids being completely eroded (Models XII and XIII in Table 1). The most spectacular example of this observed to date occurs in Drosophila, where at least thirteen protein-coding genes have been originated through DNA-mediated duplication from the autosomes to the Y chromosome [88, 164–167].

Because the Y chromosome is a nonrecombining chromosome, it is expected to degenerate over time. Nevertheless, it was found that gene duplications and their architecture within the Y chromosome play a major role in offsetting this process in some species. Examples of this can be found in humans and chimpanzees, where duplicated genes on the Y chromosome have been found in a head-to-head orientation in long palindromes [168, 169]. Further analyses of the chimpanzee male-specific region of the Y chromosome (MSY) revealed that the chimpanzee Y chromosome contains twice as many palindromes as the human MSY but also has lost some palindromes present in the MSY region in humans. This result suggests an extraordinary turnover of genes on the chimpanzee Y chromosome. Other Y or W chromosomes have also been found to contain multiple gene copies disposed in a palindrome-like structure that have been demonstrated to be undergoing concerted evolution [170–172]. Hence, gene duplication is a way of not only generating sex-specific genes but preserving the functions of these genes in nonrecombining chromosomes.

**Gene Duplications That Escape the X Chromosome**

Convergent acquisition of X to autosome retrogenes strongly suggests the existence of selective pressure acting on the fixation of these retrogenes. The retrogene *Utp14*, a gene involved in pre-rRNA processing and ribosome assembly, is a convincing example of convergence, as the recurrent emergence of this retrogenes from the same parental gene has been found in four distinct mammalian lineages [146, 173]. Potrzebowski and colleagues [16] also found two examples of recurrent emergence of retrogenes that have moved from the X-to-autosomes in mammals (*Pgk* and *Centrin*). Other studies have also revealed recurrent retroduplications from the X to autosome of in Drosophila [12]. For instance, there appears to have convergent evolution of the *Dntf-2* and *Ran *retrogenes in three independent lineages: *D. melanogaster*, *D. ananassae*, and *D. grimshawi *[69].

**Some Gene Duplications Enter the X Chromosome**

In mammals, in addition to the pattern of gene duplicates leaving the X chromosome, there is also an excess of retrogene duplicates entering the X chromosome in mammals [13]. Because retropseudogenes also show this trend, this bias is partly explained by mutational biases. However, because the trend for retropseudogenes is not as strong as it is for new retrogenes, selection was postulated to have shaped these biases [13] and with the selective pressures involved only recently being proposed [174]. A fraction of these new genes are expressed in spermatogonia or postmeiotically and support the action of selection in favor of X-linked spermatogenesis genes that are expressed before or after MSCI. For instance, *USP26* is a X-linked gene that is expressed in the spermatogonia and is evolving under positive selection [175]. Other new genes entering the X might be involved in the maternal-fetal conflict in placenta. For instance, a retrocopy of *Fth1*, a gene involved in iron metabolism, entered the X chromosome and evolved placental-specific functions.

**Autosome to Autosome Gene Duplications**

Gallach et al. [64] recently analyzed all duplicated mitochondrial genes in the nucleus of *D. melanogaster*. The authors showed that both RNA- and DNA-mediated mitochondrial gene duplications exhibit an unexpectedly high rate of relocation, and found an excess of X to autosome and also of autosome to autosome duplications. However, and in agreement with previous analyses, they found a significant deficiency of autosome to X duplications. They called this the avoidance-of-the-X pattern [64]. In addition, they found that the relocated genes (including those that originated from an autosomal parental gene) tend to have testes-specific expression and functions related to energy production-related functions. These genes evolve faster than their parental genes [64], and some of them (*CG18418 *and* CG6255*) have been identified as having been under positive selection [110], indicating that they have been selected for different functions in the male germline. This study suggest that although out of the X is the main pattern observed in genomes, other patterns, such as the avoidance-of-the-X pattern, may appear when a particular set of genes are analyzed independently. These observations are not explained by any of the models in Table 1, except model X, that is, SA resolution through SA allele duplication and the evolution of tissue-specific expression in the new gene.

**Tandem Duplication**

As introduced in the previous section and in the model XI in Table 1, there are sex-specific gene families that experience ongoing diversifying selection, leading to segregating variation, gene duplication and adaptive evolution. Examples of these are accessory gland proteins and female reproductive proteases [83, 152]. These genes that are often duplicated in tandem, maintain the patterns of expression of the parental genes (male or female tissue-specific expression), and exhibit recurrent pseudogenization, copy number variation, and gene conversion, revealing the strength and the changing direction of selection. Gene duplication is an important mechanism for diversifying preexisting sex-specific functions.

## 5. Conclusions

The advent of high-throughput technologies has allowed evolutionary biologists to analyze sexual dimorphisms in expression at the genomic level. Microarrays have been primarily used for these analyses, although next generation sequencing technologies (i.e., RNA-seq) are now being successfully used and will probably allow more direct, homogeneous, and conclusive results for many types of analyses. Despite the associated technical problems, important conclusions regarding the origin and evolution of sex-biased and sex-specific expression can be extracted from microarray analyses. A significant fraction of genes exhibit biased expression in one sex, and male bias is more common than female bias. In addition, male-biased genes tend to be expressed in a tissue-specific manner, generating most of the sex-biased expression in testis. In addition, male-biased genes evolve in a significantly different fashion from female-biased and unbiased genes: male-biased genes normally evolve increased levels of expression in this sex, the ratio interspecific divergence to intraspecific divergence is high compared with female-biased, and unbiased genes and male-biased genes have higher evolutionary rates than other genes. These observations provide evidence that male-biased genes are an important source of evolutionary change, are under strong selective pressures, and contribute greatly to sexual dimorphism.

Sex-biased genes are not randomly distributed across genomes. Instead, demasculinization of the X chromosome (i.e., a significant absence of male-biased genes in this chromosome in most cell types) is observed in many species and conflicting, inconclusive results, have been published regarding the Z chromosome. Three main hypotheses (and their derivatives) have been suggested to explain this: dosage compensation (DC), meiotic sex chromosome inactivation, (MSCI) and sexual antagonism (SA). Each one of these hypotheses may apply to a big fraction of the sex-biased genes in a given lineage, but, in another lineage, the particular hypothesis might have low predictive power. Many studies in the last few years have emphasized that gene duplication is likely an important mechanism for the evolution of sex-biased gene expression in situations where the parental genes are constrained by genomic clashes, DC or MSCI, adaptive conflict, or sexual conflict (SA). Recent study of trends in gene relocation and the evolutionary features of the new genes suggest that gene duplication might be an important mechanism for the resolution of intralocus sexual conflict. In particular, we emphasize models that may deal with many of the unexplained observations reported for DC, MSCI, and the classical model of SA resolution. Upcoming of new genome sequences from organisms with XY and ZW systems, better studies of gene gains and losses, increased availability of more precise and unbiased expression data, and the functional analyses of the relevant genes will permit the explanatory power of each hypothesis to be measured in every lineage.

Indeed, the same forces that are driving fast evolution of sex-related proteins, such as sexual selection and sexual antagonism, might also promote the creation of new sex-biased genes through gene duplication and help explain the young age of these genes and their rapid turnover.

## Figures and Tables

**Table 1:** Models proposed to explain the location of sex-biased genes in genomes with heteromorphic sex chromosomes.

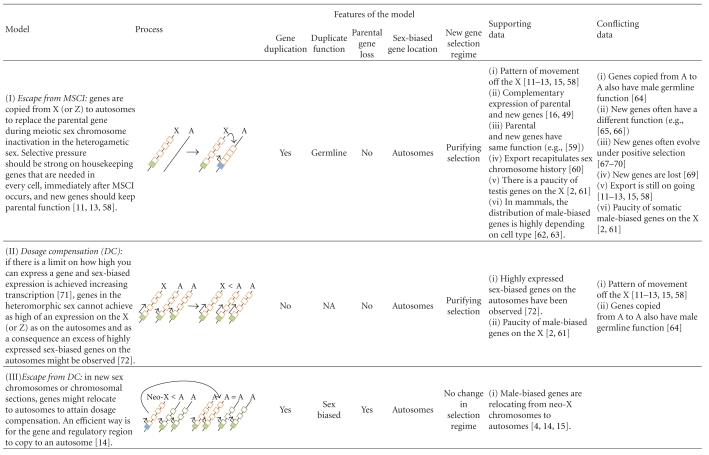 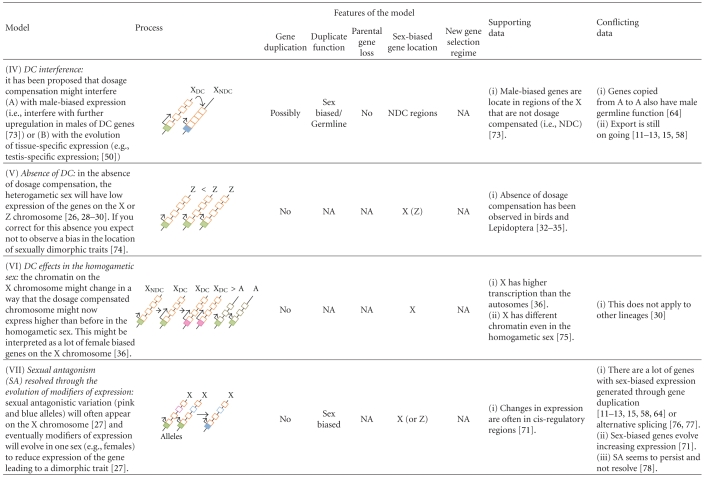 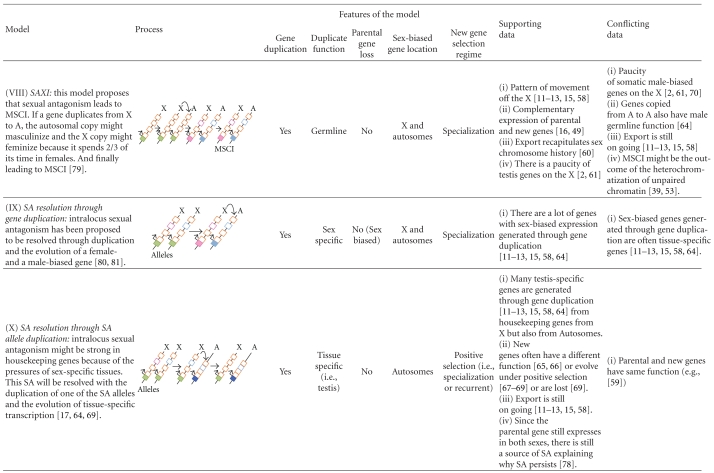 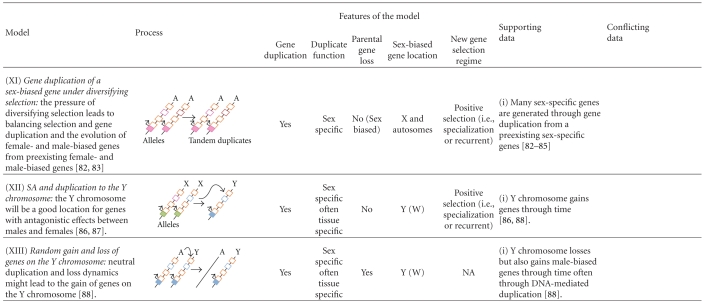

Straight arrows indicate steps and level of expression if located in the transcription start site (TSS). Length of the arrow at TSS represents the level of transcription of the gene. Curved arrows indicate duplication or relocation. Cis-regulatory regions of genes are shown in filled boxes and exons in open boxes. Green and orange refer to transcription and function in both sexes. Pink refers to female transcription or specialization of an exon. Light blue refers to male transcription or specialization of an exon. Bright blue refers to tissue-specific transcription.

**Table 2:** Summary of microarray studies data for sex-biased expression in nonvertebrate animals and male versus female contributions to sex-biased expression.

Species		Approach	Tissue	Cut-off	Sex-biased Percentage	Male/Female	References
*Drosophila*	*D. melanogaster*	cDNA Microarray (4,000 cDNAs)	Whole body	Twofold*∖* Fourfold	>50%*∖*NA	Females*∖*Males	Ranz et al. [3]
		FlyGEM Microarray	Gonads and whole body	Twofold	17%	Males (64%)	Parisi et al. [61]
		Affymetrix Microarray (18,800 transcripts)	Whole body	FDR ≤ 0.01*∖* Twofold	88%*∖*25%	~Equal contribution from males and females	Ayroles et al. [98]
	*D. pseudoobcura*	Oligo Microarray	Whole body	Twofold	42%	Males (64%)	Jiang and Machado [95]
	*Seven Drosophila*	Oligo Microarray	Whole body	FDR ≤ 0.01	13% to 32%	Male mostly (5 out of 7 species)	Zhang et al. [97]
	*D. simulans*	cDNA Microarray (4,000 cDNAs)	Whole body	Twofold*∖* Fourfold	>50%*∖*NA	Females*∖*Males	Ranz et al. [3]

*Tribolium castaneum*		cDNA Microarray (98% of cDNAs)	Whole body	FDR ≤ 0.01	20%	Females (58%)	Prince et al. [36]

*Caenorhabditis elegans*		Microarray (94% of the gene set)	Whole bodies and mutants lacking germline	Twofold	29%	Hermaphrodites	Reinke et al. [5]

*Anopheles gambiae*		Affymetrix Microarray	Whole bodies	Fourfold	10%	Females (71%)	Hahn and Lanzaro [101]
